# Calculation of hole spacing and surrounding rock damage analysis under the action of in situ stress and joints

**DOI:** 10.1038/s41598-022-27017-w

**Published:** 2022-12-25

**Authors:** Xingchao Tian, Tiejun Tao, Xia Liu, Jian Jia, Caijin Xie, Qianxing Lou, Qingzhi Chen, Zhenhua Zhao

**Affiliations:** 1 College of Civil Engineering, Guizhou University, Guiyang, 550025 China; 2 College of Mining, Guizhou University, Guiyang, 550025 China; 3 Northwest Engineering Co., Ltd. of CCCC First Highway Engineering Co., Ltd., Xi’an, 710000 China

**Keywords:** Civil engineering, Petrology

## Abstract

In situ stress and joints have a significant impact on the propagation and attenuation pattern of blast stress waves, and they are two important factors that must be considered for tunnel blasting hole network deployment. This paper proposes a blast stress wave attenuation equation and a peripheral hole distance calculation method under the combined action of in situ stress and joints. First, the static and dynamic parameters of the jointed slate are obtained by drilling core samples in the field and conducting indoor tests. Next, considering the geometric and physical attenuation of the blast stress wave, the attenuation formula of the blast stress wave under the combined action of in situ stress and joints is derived. Based on the theory of the combined action of stress waves and explosive gas, a formula for calculating the peripheral hole distance that integrates the effects of in situ stress, joints, and tensile strength of the rock body is proposed. Finally, LS-PREPOST software is used to analyze the damage to the surrounding rock, verified by an on-site blasting test. The results show that the blast stress wave attenuation formula proposed in this paper can accurately predict the stress wave peak value under the combined action of in situ stress and joints. Combining the geological conditions and blasting parameters of the Bayueshan Tunnel study section, the optimal peripheral hole spacing is calculated to be 45 cm. The average over-excavation value of the grade IV surrounding rock is controlled within 22 cm and the over-consumption of concrete per linear meter is controlled within 100% using the peripheral hole layout method and the hole network layout parameters proposed in this paper. The research results provide a reference for the control of over-excavation and under-excavation in large-section tunnel blasting.

## Introduction

The drilling and blasting method has the advantages of economic efficiency and is currently the main method for the geotechnical excavation of highway and railway tunnels. However, in the tunnel drilling and blasting method construction process, the problem of over-excavation and under-excavation is always a top-priority scientific problem that needs to be solved. Over-excavation and under-excavation not only affect the quality of tunnel blasting and increase tunnel support structure requirements^1,2^ but also raise operating costs and reduce construction progress. Therefore, it is of great importance for safe and efficient tunnel blasting to propose effective measures to control over-excavation and under-excavation.

A reasonable arrangement of peripheral holes is an effective means to control the over-excavation and under-excavation in tunnel blasting. Wang et al.^3^ established a numerical analysis model of tunnel smooth blasting using LS-DYNA software. By comparing the results of numerical simulation and field monitoring, it was shown that the spacing of peripheral holes should be mainly considered in the massive structure and the fractured structure. Paul and Peter^4^ analyzed the causes, hazards, and control measures of tunnel over-excavation and under-excavation caused by the drilling and blasting method through a model test system, and proposed a reasonable peripheral hole design scheme. Adel et al.^5^ established a tunnel over-excavation prediction model considering factors such as tunnel cross-sectional area and peripheral hole parameters. Yin et al.^6^ proposed a drilling method combining long and short holes in the peripheral holes, which effectively solved the problem of over-excavation and under-excavation. Liu et al.^7^ indicated that the peripheral hole parameters should be adjusted in time according to changes in the surrounding rock conditions. Huang et al.^8^ studied the application of a double-layer peripheral hole retaining wall blasting design in the blasting engineering of the soft and broken surrounding rock, and optimized the peripheral hole layout and charge structure. Based on the principle of smooth blasting, Man and Liu^9^ studied the blast hole arrangement scheme of peripheral holes in the case of empty hole spacing. Man et al.^10^ focused on the effect of peripheral hole spacing on the effect of smooth blasting.

However, a rock mass in nature exhibits anisotropy due to the existence of structural planes such as joints and fissures^11^ (1988), when the blast stress wave propagates to these structural surfaces, multiple reflections occur, resulting in the attenuation of the blast stress wave. Hyongdoo et al.^12^ proposed the over-excavation resistance coefficient (ORF) based on the relationship between the over-excavation measured values and geological parameters, indicating that the discontinuity factor has the greatest impact on blasting and over-excavation. Zhao and Cai^13^, Cai et al.^14^, and Zhao et al.^15,16^ studied the propagation and attenuation pattern of stress waves after vertically traversing groups of parallel joints. Li et al.^17–19^ derived the propagation equation of multiple parallel joints with the oblique incidence of stress waves. Perino et al.^20^ derived the propagation and attenuation pattern of stress waves in cross joints. Chai et al.^21^ derived the propagation equation of plane P-waves in a rock mass with two intersecting joints.

At the same time, the rock surrounding a tunnel is already in a certain initial stress state before it is subjected to a blast load. When the initial stress state changes, the micropores inside the rock mass evolve, affecting the propagation of blast stress waves. Mandal and Singh^22^ believed that the initial stress field of the surrounding rock had a great influence on the blast load. Mandal et al.^23^ indicated that the influence of initial in situ stress and stress redistribution should be considered during tunnel excavation, and based on this, an empirical formula for stress wave attenuation in media was proposed. Zheng ^24^ carried out a three-dimensional high in situ stress rock mass ultra-deep hole blasting test, and concluded that the initial in situ stress has an inhibitory effect on the expansion of blast cracks. Zhang et al.^25^ studied the damage to surrounding rock under blast load, showing that in situ stress has an inhibitory effect on the blast tension effect. Li et al.^26^ studied the propagation and attenuation pattern of elastic waves in a deeply fractured rock mass through model tests. Li et al.^27^ examined the propagation pattern of stress waves with the vertical incidence of multiple parallel jointed rock masses under different in situ stresses based on indoor model tests. Fan et al.^28^ analyzed the propagation pattern of a one-dimensional stress wave vertically incident on a single joint under the action of in situ stress. Liu and Xu^29^ established the differential equation of the motion of rock mass under the action of an explosion load and under the action of in situ stress, and completed the numerical simulation of a rock mass explosion under the action of in situ stress through the implicit–explicit continuous solution. Fan et al.^30^ studied the propagation pattern of stress waves in the rock medium under different initial stresses based on a numerical analysis method. Zhang et al.^31^ researched the propagation pattern of stress waves and the action mechanism of joint surfaces under the combined action of in situ stress and joints.

At present, most of propagation theories about stress waves in jointed rock masses treat the stress wave as a plane wave, and research on the attenuation pattern of cylindrical waves under the combined action of in situ stress and joints is rarely reported. In this paper, we took the Tongliang–Anyue Expressway Bayueshan Tunnel as the research background, drilled rock samples in the field, and conducted laboratory tests to obtain the static and dynamic parameters of the jointed slate. Considering the geometric and physical attenuation of the blast stress wave at the same time, the attenuation formula of the blast stress wave under the combined action of in situ stress and joints is proposed. Based on the combined action of stress wave and explosive gas theory, a calculation formula for peripheral hole spacing is proposed that comprehensively considers the effects of in situ stress, joints, and rock mass tensile strength. LS-PREPOST software is used to establish a single-hole blasting numerical analysis model of an in situ stress-jointed rock mass, and to verify the correctness of the explosion stress wave attenuation formula. We established numerical analysis models for tunnel blasting with peripheral hole spacings of 40 cm, 45 cm, 50 cm, and 55 cm, respectively, and compared the damage depth and peak vibration velocity (*PPV*) of the retained rock mass to verify the rationality of the calculation formula for the peripheral hole spacing. We conducted field blasting tests based on the theoretical derivation and numerical analysis results to provide a reference for the control of over-excavation and under-excavation in large-section tunnel blasting.

## Materials and methods

### Engineering background

**Figure 1:**
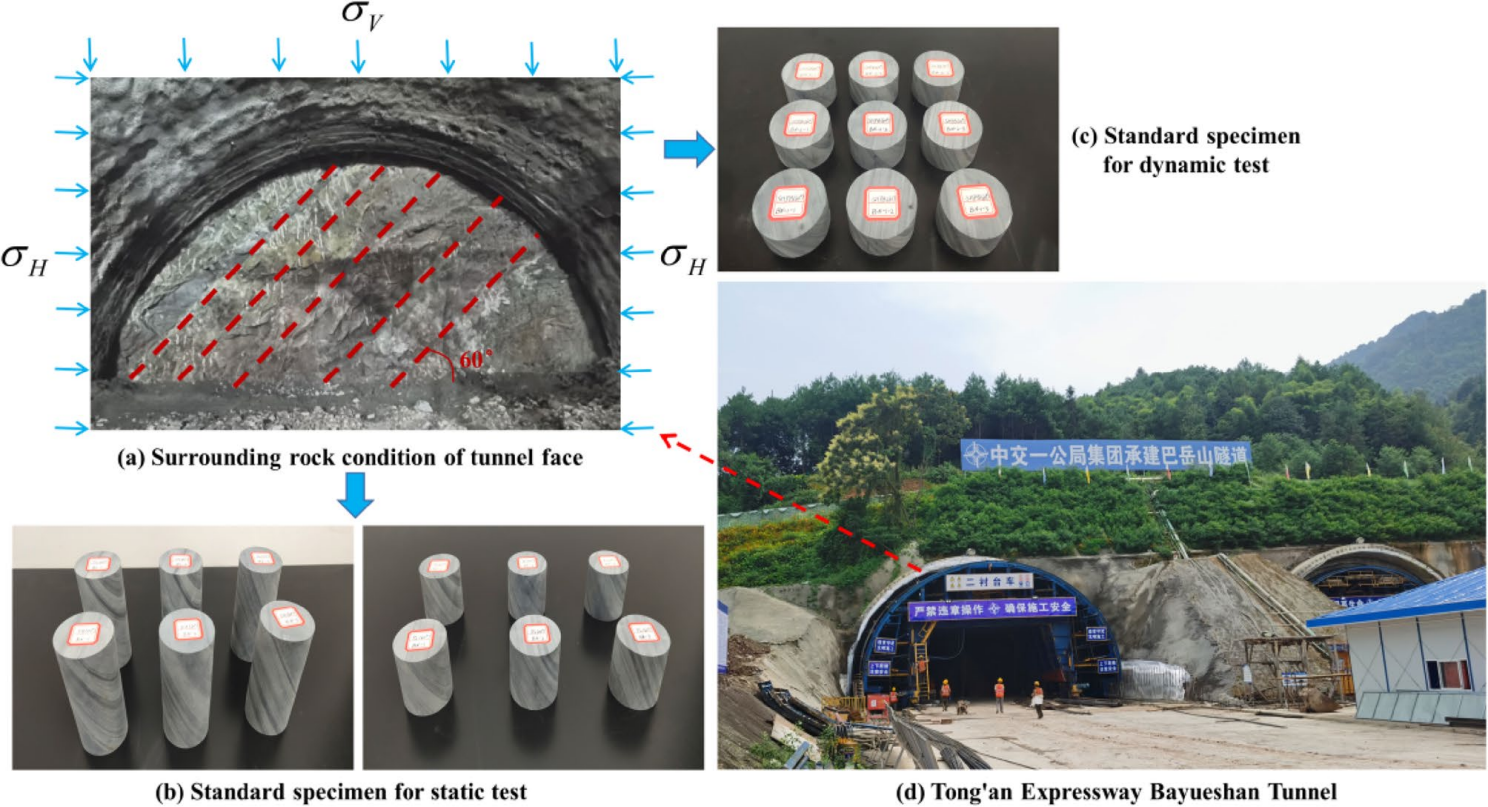
Engineering background. (**a**) surrounding rock condition of tunnel face; (**b**) standard specimen for static test; (**c**) standard specimen for dynamic test; (**d**) Tong’an Expressway Bayueshan Tunnel.

The research takes the ZK13 + 760 ~ ZK13 + 940 section of the Tongliang–Anyue Expressway Bayueshan Tunnel as the engineering background. The total length of the research section is 180 m, the average burial depth is 56 m, the rock mass integrity coefficient *K*_*v*_ is 0.68, and the revised basic quality index of the surrounding rock (BQ) is 321, which is classified as grade IV. The upper layer of the tunnel site is covered with residual slope deposits (Q^el+dl^), containing gravelly powder clay, with 35–40% gravel content and 0.5–2 cm particle size. The underlying bedrock of the tunnel site is tuffaceous slate of the second section (Ptbnbf^2^) of the Fanzhao Formation of the upper Banxi Group in the Yuangu realm. According to the characteristics of rock joints and fissure development, hardness and integrity, it is divided into two layers: a strong weathering layer (4.5–24.5 m) and a medium weathering layer. Figure 1 shows the rock surrounding the tunnel face in the study section. The lithology of the surrounding rock is mainly slate, and there are many joints and fissures at 60° to the horizontal plane. To obtain the static and dynamic parameters of slate under the combined action of vertical load *σ*_*V*_ and horizontal load *σ*_*H*_, we drilled core rock samples on site and made standard slate samples with 60° joint fissures, and we carried out uniaxial compression, triaxial compression, Brazilian splitting, and impact dynamics tests.

### Determination of slate static parameters

**Figure 2:**
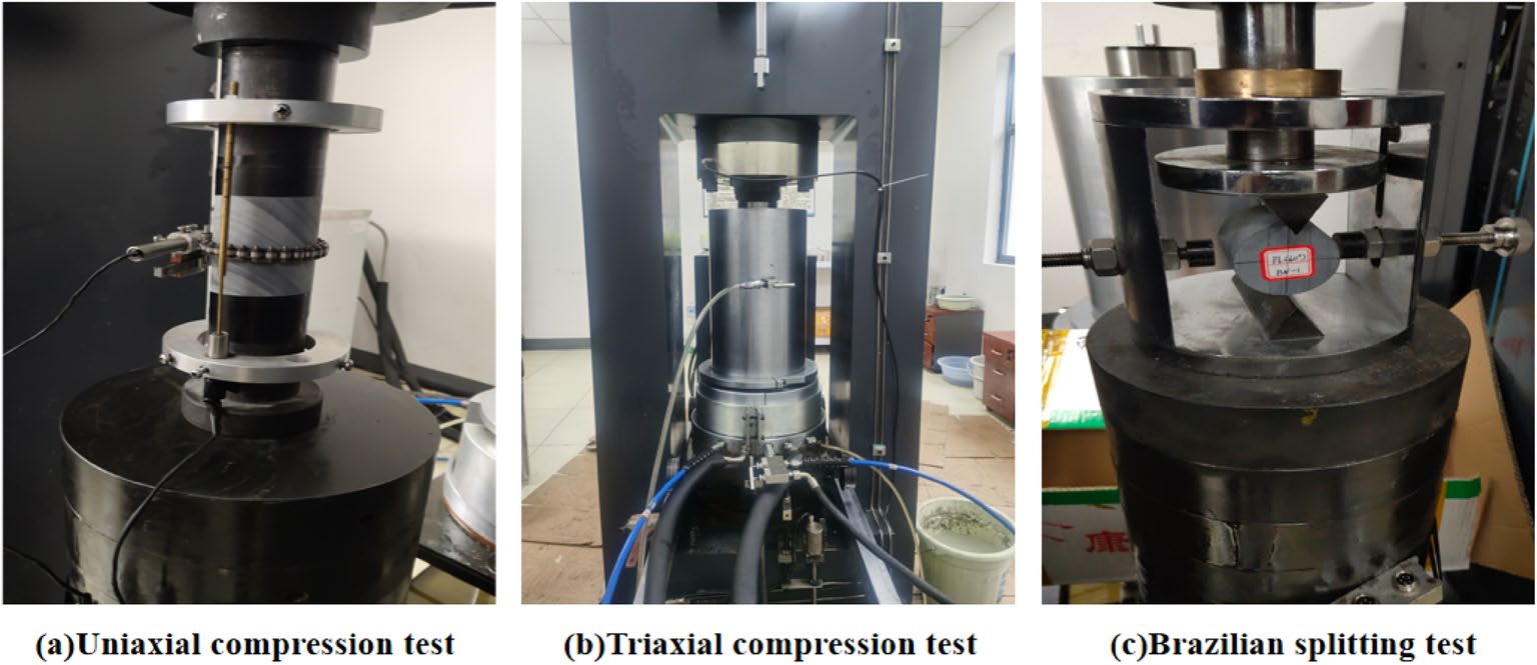
Static test. (**a**) Uniaxial compression test; (**b**) triaxial compression test; (**c**) Brazilian splitting test.

We measured the diameter and the height of the rock samples with vernier calipers, and weighed the rock samples with an electronic balance. The densities of the nine rock samples were 2746 kg/m^3^, 2747 kg/m^3^, 2744 kg/m^3^, 2749 kg/m^3^, 2754 kg/m^3^, 2778 kg/m^3^, 2735 kg/m^3^, 2758 kg/m^3^, and 2752 kg/m^3^, with an average density of 2752 kg/m^3^. The uniaxial compression test, triaxial compression test, and Brazilian splitting test were carried out on the microcomputer-controlled electro-hydraulic servo rock triaxial testing machine TAJW-2000, as shown in Fig. 2.

**Figure 3:**
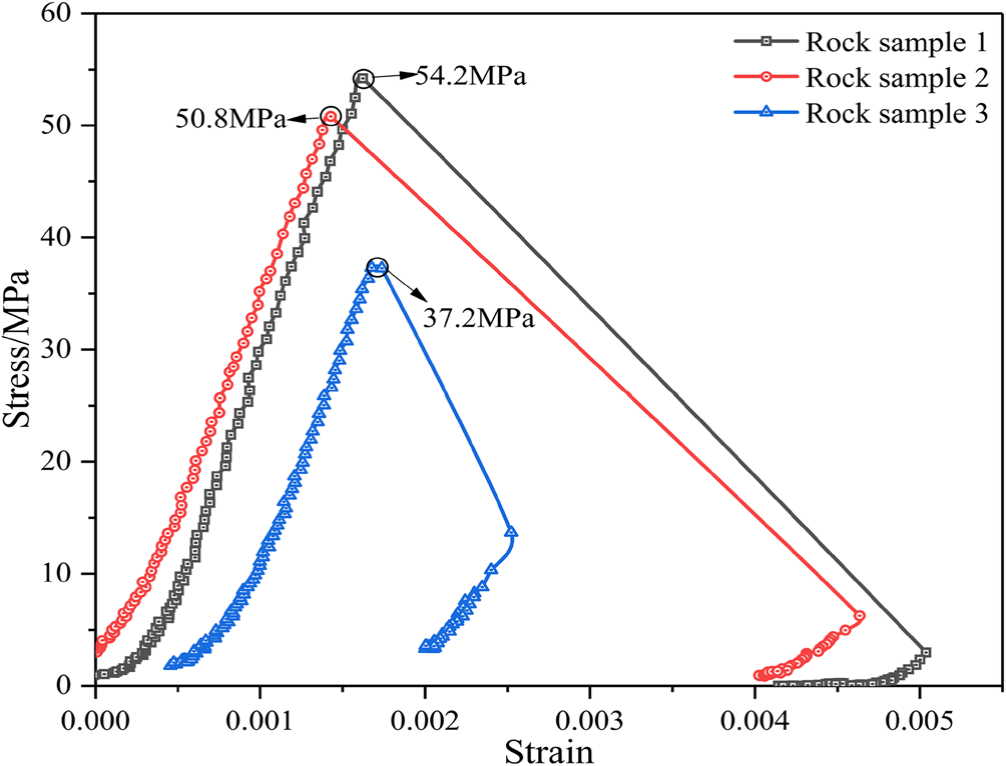
Uniaxial compressive stress–strain curve.

In the uniaxial compression test, a preload of 0.2 KN was first applied and then converted to displacement loading with a loading rate of 0.12 mm/min after the instrument was stabilized. The load was continuously applied until the specimen was damaged, and followed by immediate unloading. In the uniaxial compression test process, shown in Fig. 2a, the displacement change process of the rock specimen was collected by an axial displacement transducer and a circumferential displacement transducer, with the stress–strain curve shown in Fig. 3. The uniaxial compressive strengths of the three rock samples were 54.2 MPa, 50.8 MPa, and 37.2 MPa, respectively, and the average uniaxial compressive strength *f*_*c*_ was 47.4 MPa. In Fig. 1, the slate is subjected to the combined action of the vertical load *σ*_*V*_ and the horizontal load *σ*_*H*_, which is two-way loading. The literature Huang^32^ shows that the ratio *β* of bidirectional compressive strength *f*_*bc*_ to uniaxial compressive strength *f*_*c*_ is 1.493*f*_*c*_^– 0.0634^. Therefore, the bidirectional compressive strength *f*_*bc*_ of slate is 55.4 MPa. The elastic moduli of the three rock samples were 40.2 GPa, 38.6 GPa, and 35.3 GPa, respectively, with an average elastic modulus of 38.03 GPa. The Poisson’s ratios of the three rock samples were 0.23, 0.28, and 0.29, respectively, with an average Poisson’s ratio of 0.27.

In the Brazilian splitting test, a linear load was applied in the diameter direction of the cylindrical rock specimen with a loading rate of 0.15 mm/min until the specimen was damaged. The Brazilian splitting test process is shown in Fig. 2c. The tensile strength $\sigma_{t}$ of the rock sample can be calculated as follows:

1 $$ \sigma_{t} = \frac{2P}{{\pi DL}} $$

where *P* is the load corresponding to the splitting failure of the rock sample, and *D* and *L* represent the diameter and height of the rock sample, respectively. The loads of the three rock samples at failure were 43.5 kN, 41.6 kN, and 56.5 kN, respectively. According to Eq. (1), the tensile strength of the three rock samples were 11.1 MPa, 10.6 MPa, and 14.4 MPa, respectively, and the average tensile strength of the rock samples was 12.03 MPa.

### Determination of slate dynamic parameters

**Figure 4:**
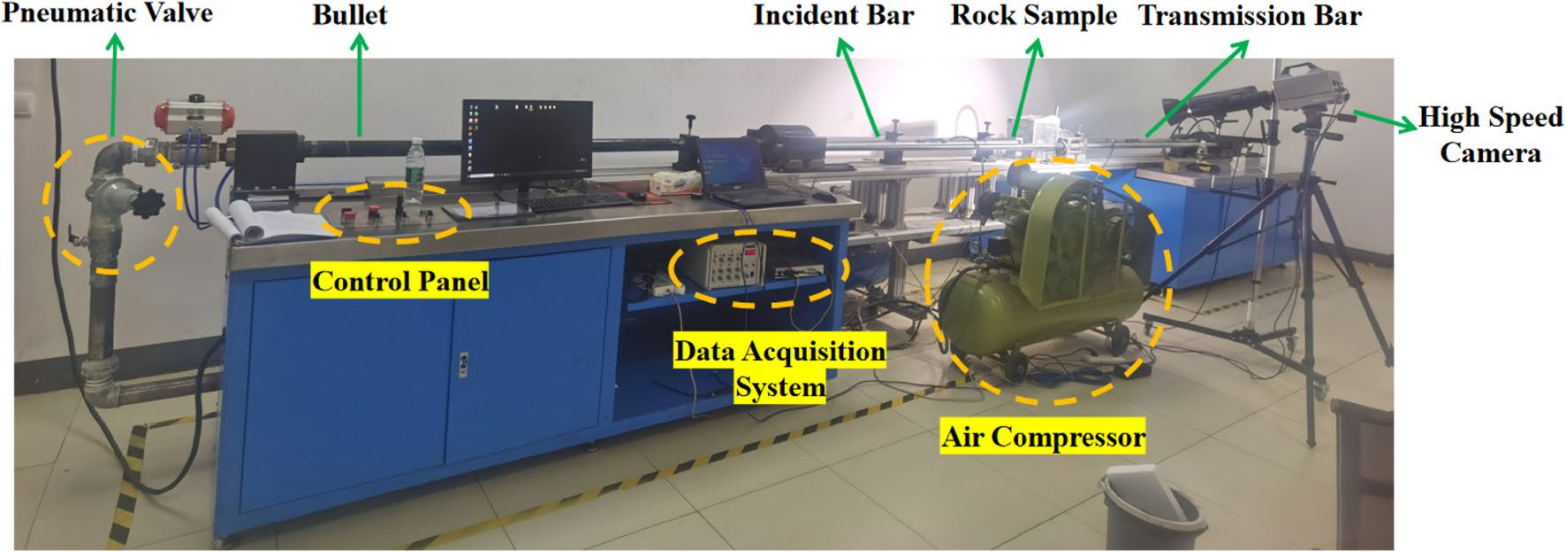
SHPB test system.

The slate impact dynamic test was carried out using the separate Hopkinson pressure bar test system ALT100 developed by Archimedes Industrial Technology Co., Ltd., as shown in Fig. 4. The diameters of the impact bar, incident bar, and transmission bar were all 50 mm, and their lengths were 400, 2000, and 2000 mm, respectively. The density of the pressure bar was 7.81 g/cm^3^, the elastic modulus was 210 GPa, the Poisson's ratio was 0.28, and the longitudinal wave velocity was 5410 m/s. The data acquisition system comprised strain gauges pasted on the pressure bar, a wheatstone bridge (strain gauge junction bridge box), super dynamic strain gauges, and a high-speed acquisition system.

Based on the assumption of one-dimensional stress waves and dynamic balance, the dynamic stress $\sigma \left( t \right)$ and dynamic strain $\varepsilon \left( t \right)$ can be accurately calculated using the “two-wave method”, with the incident strain signal $\varepsilon_{I} \left( t \right)$, the reflected strain signal $\varepsilon_{R} \left( t \right)$, and the transmitted strain signal $\varepsilon_{T} \left( t \right)$ measured on the pressure bar. The calculation method is as follows:

2 $$ \begin{aligned}   \sigma \left( t \right) &  = \frac{{A_{0} E}}{{2A}}\varepsilon _{T} (t) \\    \varepsilon \left( t \right) &  =  - \frac{{2C}}{L}\int_{{\text{0}}}^{T} {\left[ {\varepsilon _{R} (t)} \right]dt}  \\  \end{aligned}   $$

where *A* and *L* are the cross-sectional area and length of the sample, respectively; *E* and *A*_*0*_ represent the elastic modulus and cross-sectional area of the pressure bar, respectively; and *C* denotes the longitudinal wave velocity of the pressure bar.

**Figure 5:**
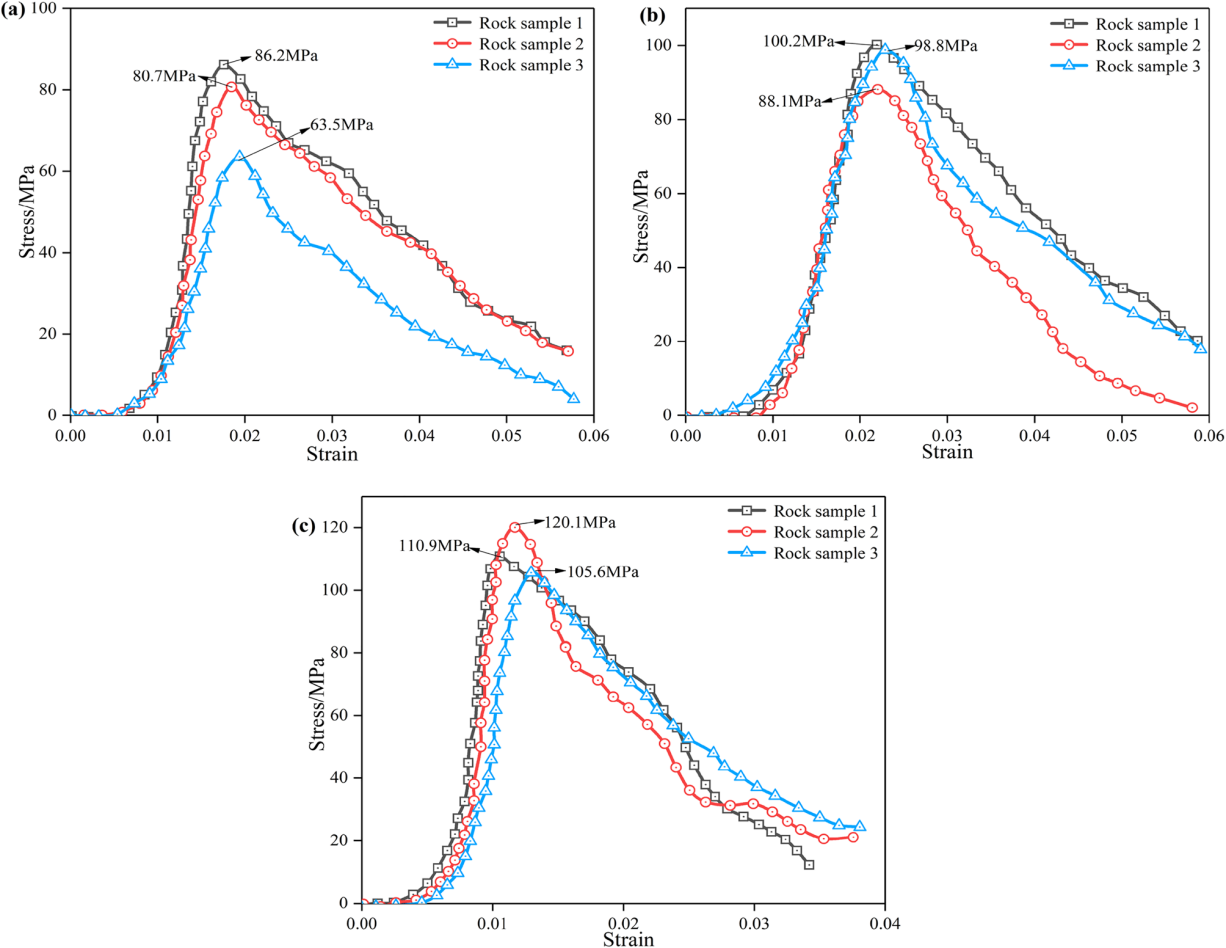
Impact compressive stress–strain curves: (**a**)–(**c**) are impact pressures of 0.15 MPa, 0.2 MPa, and 0.3 MPa, respectively.

Impact compression tests were conducted on the slate samples under the impact pressure of 0.15 MPa, 0.2 MPa, and 0.3 MPa, respectively, and the impact compression stress–strain curve was drawn through Eq. (2), as shown in Fig. 5. The average peak stress of slate under 0.15 MPa, 0.2 MPa, and 0.3 MPa impact pressures was 76.8 MPa, 95.7 MPa, and 112.2 MPa, respectively. The dynamic tensile strength of rock has little sensitivity to the loading strain rate, and it can be considered that the dynamic tensile strength of rock is the same as the uniaxial tensile strength. The mechanical parameters of the 60° jointed slate are shown in Table 1.

**Table 1:** Mechanical parameters of 60° jointed slate.

Density /(kg m^−3^)	Elastic modulus /GPa	Poisson's ratio	Uniaxial compressive strength/MPa	Biaxial isostatic strength/MPa	Uniaxial tensile strength/MPa	Dynamic tensile strength/MPa
2752	38.03	0.27	47.4	55.4	12.03	12.03

## Determination of the peripheral hole spacing

### Attenuation pattern of cylindrical wave in jointed rock mass under different confining pressures

In the area near the blasting, the stress wave generated by the blasting of the blasthole usually propagates outward in the form of a cylindrical stress wave (two-dimensional wave), and its wavefront series of cylindrical surfaces take the central axis of the blasthole as the axis. Cylindrical waves in intact rocks undergo both geometric and physical attenuation with increasing propagation distance^33^, where geometric attenuation is the attenuation due to the increase in the spatial distribution of stress wave energy. The stress on the wavefront of the cylindrical wave and the vibration velocity of the particle are both attenuated by $1/\sqrt r$^34^, where *r* is the distance between the wavefront and the wave source. The particle vibration velocity *v*_*r*_ on the wavefront distanced from the wave source *r* is

3 $$ v_{r} = v_{0} \sqrt {r_{0} /r} $$

where *r*_*0*_ and *v*_*0*_ are the radius of the initial wavefront of the cylindrical wave and the vibration velocity of the particle, respectively. The physical attenuation of cylindrical waves in intact rocks is caused by the friction effect on the surface of microcracks inside the propagating medium^35^, and the particle vibration velocity on the cylindrical wave wavefront decays negatively exponentially with the increase in propagation distance:

4 $$ v_{r} = v_{0} \cdot e^{ - \alpha r} $$

where $\alpha$ is the physical attenuation coefficient of the cylindrical wave.

In summary, considering both physical attenuation and geometric attenuation, the propagation attenuation equation of cylindrical waves in intact rock is

5 $$ v_{r} = v_{0} \left( {\sqrt {r_{0} /r} + e^{ - \alpha r} } \right) $$

According to the stress wave propagation theory, radial stress $\sigma_{r}$ at a certain point caused by the propagation of the cylindrical stress waves in the rock mass is

6 $$ \sigma_{r} = \rho c_{r} v_{r} $$

It can be seen from the elastic mechanics that, without considering the tangential strain $\varepsilon_{\theta }$, it can be considered that the radial stress $\sigma_{r}$ and the radial strain $\varepsilon_{r}$ at a certain point in the rock body approximately satisfy the following relationship:

7 $$ \sigma_{r} \approx E_{d} \cdot \varepsilon_{r} $$

Combining Eqs. (6) and (7), we can obtain

8 $$ \varepsilon_{r} = \frac{{\rho c_{r} }}{{E_{d} }}v_{r} $$

where *E*_*d*_ is the dynamic elastic modulus.

Assuming that the density, radial wave velocity, and dynamic elastic mode of the model material remain unchanged during the blasting process, the radial strain on the cylindrical wave front is approximately proportional to the particle vibration velocity. Therefore, it can be considered that the attenuation coefficient of the radial strain of the particle and the radial vibration velocity are approximately the same:

9 $$ \varepsilon_{r} = \varepsilon_{0} \left( {\sqrt {r_{0} /r} + e^{ - \alpha r} } \right) $$

**Figure 6:**
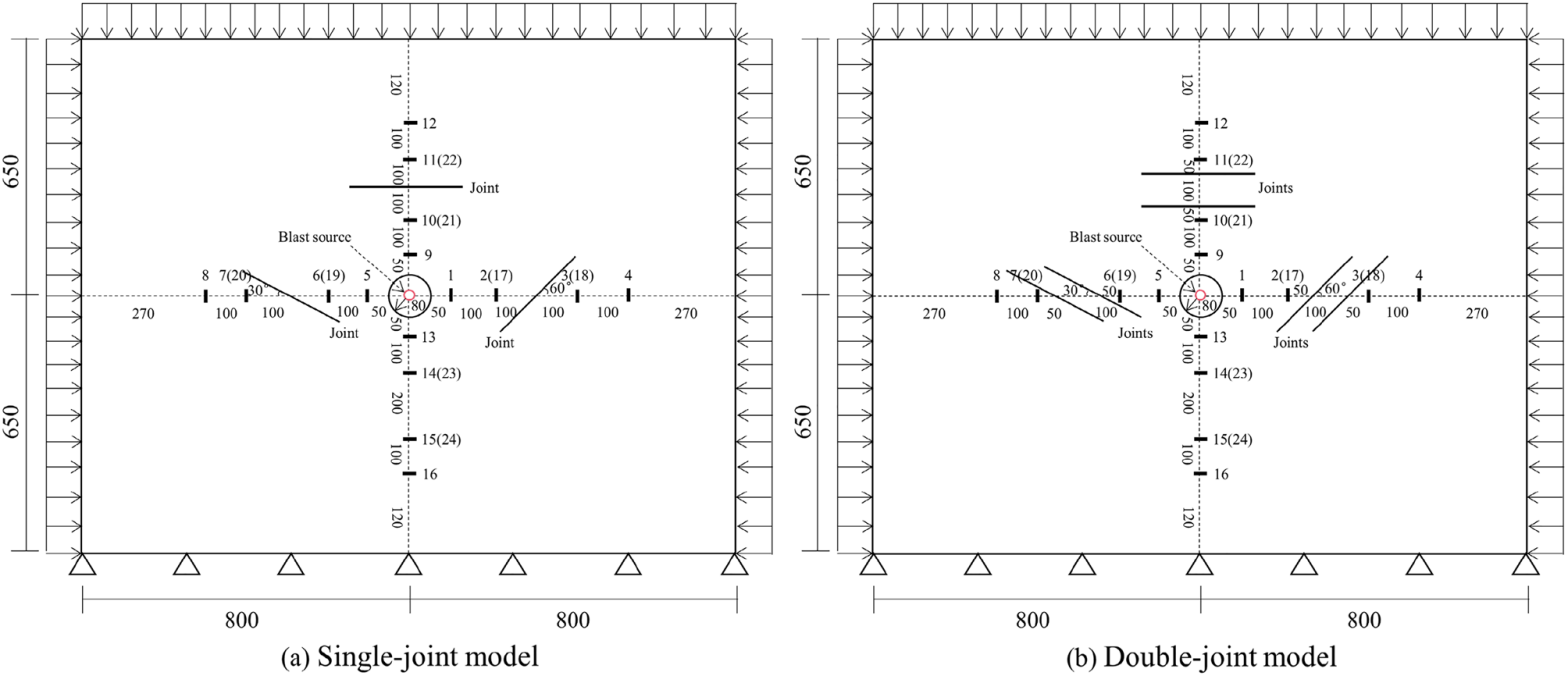
Schematic diagram of the model^36^: (**a**) single-joint model; (**b**) double-joint model.

Dong produced test blocks with single and double joints, and carried out model tests^36^. The schematic diagram of the model is shown in Fig. 6.

Through analysis of the Nos. 13–16 measuring point strain peaks under different confining pressures, the relationship between the physical attenuation coefficient $\alpha$ of the cylindrical wave in the complete rock mass and the confining pressure $\sigma$ is obtained:

10 $$ \alpha = 0.0332\sigma^{3} - 0.08596\sigma^{2} - 0.0036\sigma + 0.649 $$

Substituting Eq. (10) into Eq. (9), the attenuation pattern of the particle point radial strain when the cylindrical wave propagates in the complete rock mass under different confining pressures is obtained:

11 $$ \varepsilon_{r} = \varepsilon_{0} \left( {\sqrt {r_{0} /r} + e^{{\left( { - 0.0332\sigma^{3} + 0.08596\sigma^{2} + 0.0036\sigma - 0.649} \right)r}} } \right) $$

Substituting Eq. (11) into Eq. (7), the attenuation pattern of the particle radial stress when the cylindrical wave propagates in the complete rock mass under different confining pressures is obtained:

12 $$ \sigma_{r} = = \sigma_{0} \left( {\sqrt {r_{0} /r} + e^{{\left( { - 0.0332\sigma^{3} + 0.08596\sigma^{2} + 0.0036\sigma - 0.649} \right)r}} } \right) $$

Equation (12) is the attenuation pattern of the particle radial stress when the cylindrical wave propagates in the complete rock mass, without considering the influence of the joint on the attenuation of the radial stress. However, the rock mass is often rich in joint planes, which have a significant impact on the attenuation of radial stress at the particle point. Therefore, it is necessary to establish the attenuation relationship of the radial stress of the particle when the cylindrical wave propagates in the jointed rock mass under different confining pressures:

13 $$ \sigma_{r}^{\prime } = K\sigma_{0} \left( {\sqrt {r_{0} /r} + e^{{\left( { - 0.0332\sigma^{3} + 0.08596\sigma^{2} + 0.0036\sigma - 0.649} \right)r}} } \right) $$

where *K* is the correction coefficient of the joint, which is related to the confining pressure and the property of the joint, and its value is between 0 and 1.

**Figure 7:**
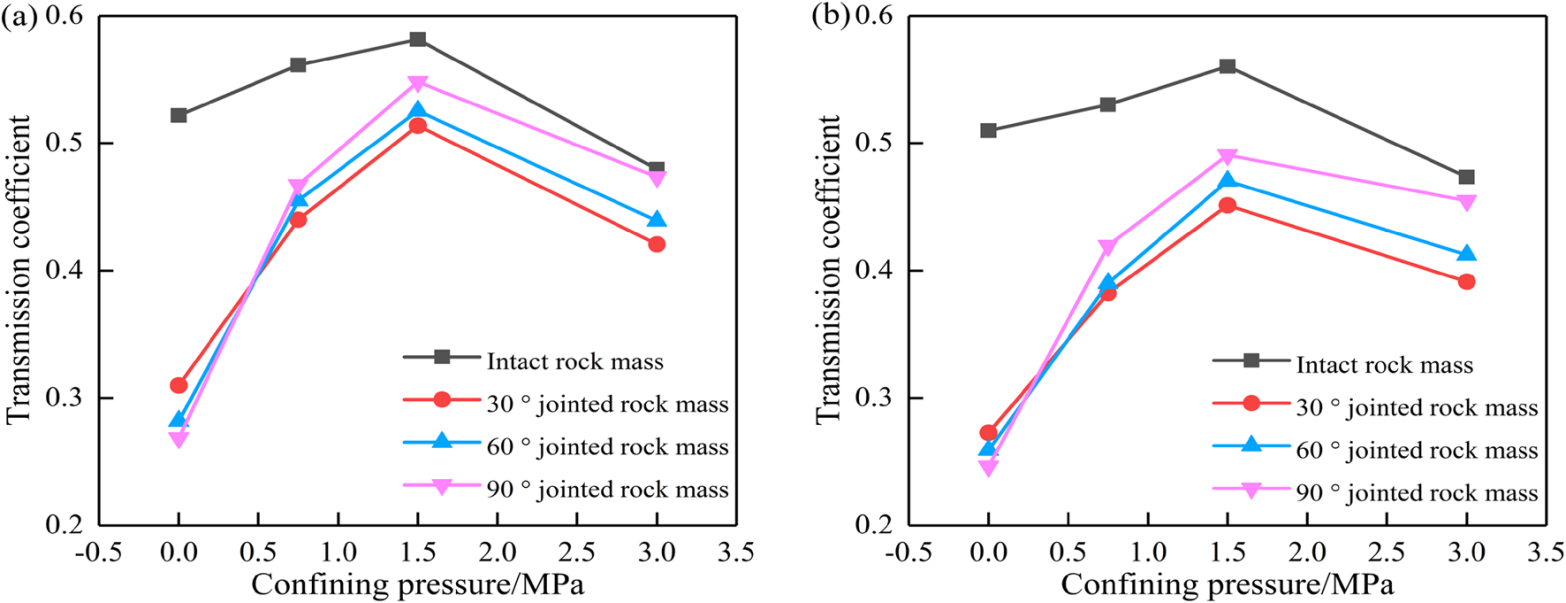
Cylindrical wave transmission coefficients under different confining pressures: (**a**) single-joint test block; (**b**) double-joint test block.

Dong^36^ set the measurement points 17–24 as the stress measurement points in the model test. The ratio of the peak values of the time history curves recorded at the stress measurement points before and after the joints with different angles is defined as the transmission coefficient of the cylindrical wave, and the variation pattern of the cylindrical wave transmission coefficient of the single-joint and double-joint test blocks under different confining pressures is obtained, as shown in Fig. 7.

**Figure 8:**
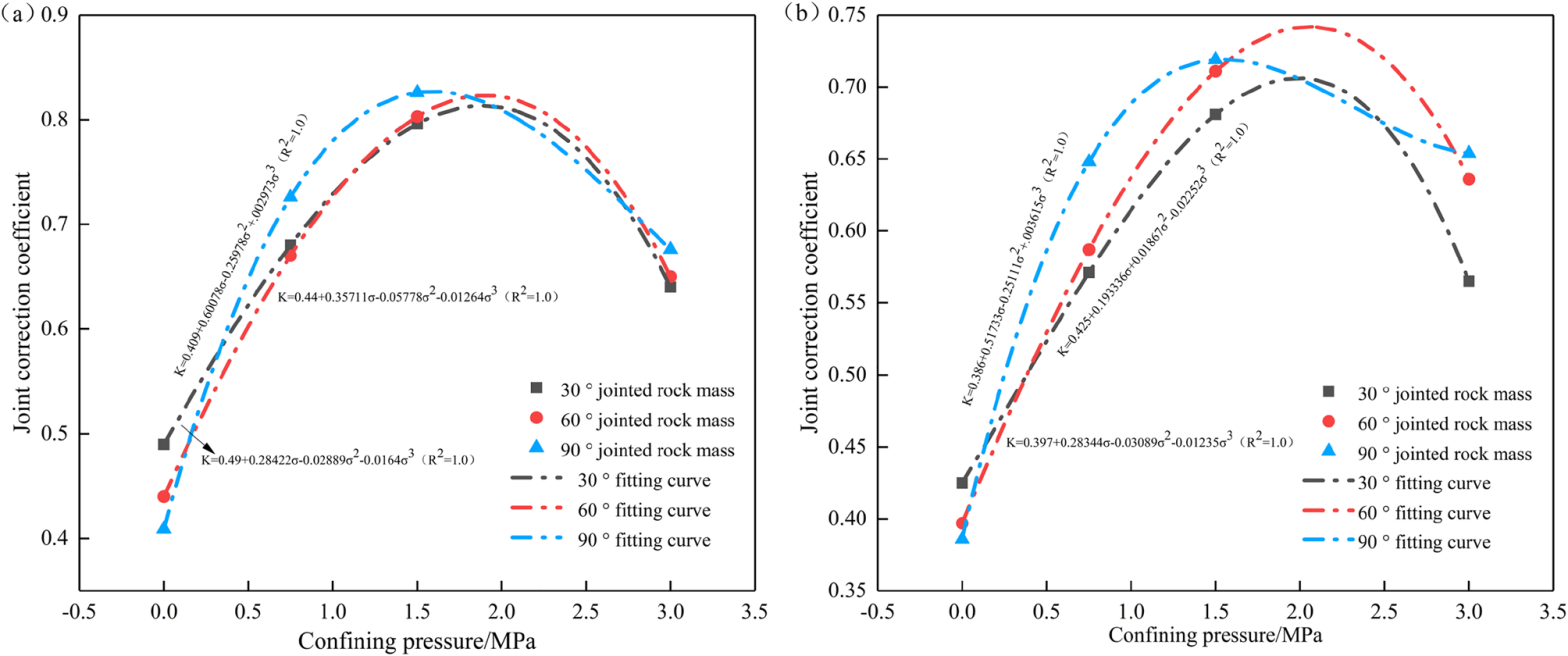
Variation of joint correction coefficient *K* with confining pressure: (**a**) single-joint test block; (**b**) double-joint test block.

The joint correction coefficient *K* is defined as the ratio of the peak value of the stress time history curve of measuring points 11, 18, and 19 to the peak value of the stress calculated by Eq. (12). Equation (12) is used to calculate the peak stress at measuring points 17–24, and the joint correction coefficient *K* can be calculated by combining with the peak data of the stress time-history curve at measuring points 17–24, as shown in Table 2. The relationship between the joint correction coefficient *K* and the confining pressure of the single-joint test block and the double-joint test block is plotted in Fig. 8.

**Table 2:** Values of joint correction coefficient *K.*

Joint inclination, K		Confining pressure			
		0 MPa	0.75 MPa	1.5 MPa	3.0 MPa
Single-joint test block	30°	0.490	0.680	0.796	0.640
	60°	0.440	0.670	0.803	0.650
	90°	0.409	0.726	0.826	0.676
Double-joint test block	30°	0.425	0.571	0.681	0.565
	60°	0.397	0.587	0.711	0.636
	90°	0.386	0.648	0.719	0.654

It can be seen from Fig. 8 that the joint correction coefficient *K* has a good correlation with the confining pressure, the joint inclination angle, and the number of joints. When there is no confining pressure, the joint correction coefficient decreases with the increase in the joint inclination angle. When the confining pressure increases from 0 to 1.5 MPa, the joints are gradually closed under the action of the confining pressure, the stiffness of the joints increases rapidly, and the attenuation of the stress wave decreases, so the joint correction coefficient *K* increases. When the confining pressure continues to increase to 3.0 MPa, micro-cracks are formed in the closed joints, and the expansion of new cracks leads to an increase in the attenuation of stress waves, so that the joint correction coefficient *K* decreases. The joint correction coefficient *K* in the double-joint test block is lower than that in the single-joint test block. The greater the number of joints, the stronger the transmission and reflection effect of the stress wave between the joints, the attenuation amplitude of the stress wave after passing through the joint group decreases, and the attenuation amplitude of the joint correction coefficient *K* decreases^37^. Therefore, this paper only analyzes the single-joint test block and the double-joint test block.

### Peripheral hole spacing calculation method

When the uncoupled continuous charge is used, the impact stress of the blast hole wall after blasting is

14 $$ P_{0} = 1/8\rho_{0} D^{2} \left( {d_{c} /d_{b} } \right)^{6} \left( {l_{c} /l_{b} } \right)^{3} n $$

where $\rho_{0}$ is the density of the explosive (kg/m^3^); *D* represents the detonation velocity of the explosive (m/s); *d*_*c*_ denotes the diameter of the cartridge (m); *d*_*b*_ refers to the diameter of the blast hole (m); *l*_*c*_ is the charge length and *l*_*b*_ is the length of the blast hole (m); and *n* depicts the pressure increase coefficient of the detonation gas impacting the hole wall, generally taking 10.

According to Eq. (13), considering the influence of in situ stress and joint properties, the radial stress and tangential stress of the particle at the distance *r* from the center of the blast hole are

15 $$ \sigma_{r}^{\prime } = KP_{0} \left( {\sqrt {r_{0} /r} + e^{{\left( { - 0.0332\sigma^{3} + 0.08596\sigma^{2} + 0.0036\sigma - 0.649} \right)r}} } \right) $$

16 $$ \sigma_{\theta } = b\sigma_{r}^{\prime } $$

where *r*_*b*_ is the radius of the blast hole (m); *b* represents the lateral pressure coefficient of the rock mass ($b = \mu_{d} /\left( {1 - \mu_{d} } \right)$); *μ*_*d*_ stands for the dynamic Poisson’s ratio; and the other symbols are the same as defined above.

The condition of tensile failure of the rock mass under the action of tangential stress $\sigma_{\theta }$ is $\sigma_{\theta } \ge \sigma_{td}$, and $\sigma_{td}$ is the dynamic tensile strength of the rock mass. Therefore, the calculation expression of the fracture circle radius *R* is

17 $$ bKP_{0} \left( {\sqrt {r_{b} /r} + e^{ - \alpha r} } \right) = \sigma_{td} $$

After determining the relevant parameters, the value of the fracture circle radius *R* can be calculated using the MATLAB program.

According to the theory of the combined action of stress wave and blasting gas, the formation of through cracks between blasting holes is due to the static pressure of the blasting gas, and the formation conditions of through cracks are

18 $$ 2r_{b} P_{b} = (D_{P} - 2R)\sigma_{td} $$

where *D*_*P*_ is the peripheral hole spacing (m); and *P*_*b*_ represents the pressure when the blast hole is filled with explosive gas (Pa).

Based on the theory of entropy expansion, the pressure *P*_*b*_ when the blast hole is filled with explosive gas is

19 $$ P_{b} = (P_{a} /P_{k} )^{k/h} (V_{c} /V_{b} )^{k} P_{k} $$

where *P*_*a*_ is the explosion pressure (Pa); *P*_*k*_ stands for the critical pressure during the expansion of the explosion gas (taking 100 MPa); *V*_*c*_ and *V*_*b*_ represent the volume of the cartridge and the blast hole, respectively (m^3^); *k* denotes the adiabatic coefficient of the explosive; and *h* refers to the isentropic coefficient of the explosive (*k* = *h* = 3.0).

From Eqs. (17)–(19), the calculation expression of the peripheral hole spacing under the action of in situ stress and joints can be obtained:

20 $$ D_{P} = 2\left( {R + r_{b} P_{b} /\sigma_{td} } \right) $$

According to the blasting manual, when *D*_*P*_ = 0.8 W, the blasting effect is better, and the value of the minimum resistance line *W* can be calculated.

### Verification of the attenuation formula

**Figure 9:**
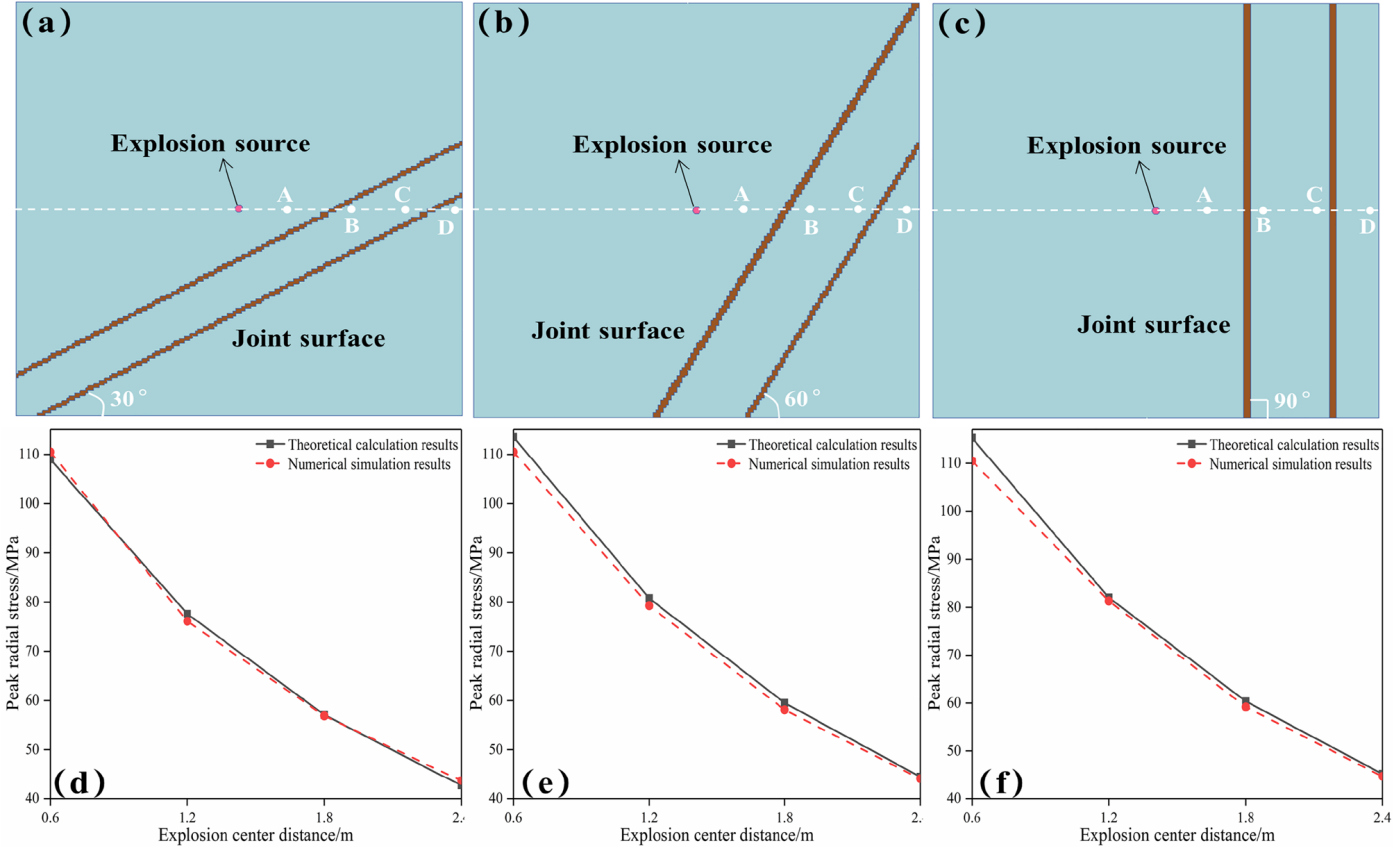
Verification of the explosion stress wave attenuation formula: (**a**)–(**c**) the three-dimensional numerical calculation models under different joint inclination angles; (**d**)–(**c**) the comparison between the theoretical calculation results and the numerical simulation results.

Based on the original blast stress wave attenuation formula, this paper proposes a new blast stress wave attenuation formula by considering the combined effect of in situ stress and joints. To verify the correctness of Eq. (15), LS-PREPOST software was used to establish a three-dimensional numerical calculation model of the double joint under 1.5 MPa in situ stress, as shown in Fig. 9a–c. In Fig. 9a–c, the in situ stress is 1.5 MPa, and the physical attenuation coefficient *α* is 0.56224. The dip angles of the joints are 30°, 60°, and 90°, and the joint correction coefficients *K* are 0.681, 0.711, and 0.719, respectively. To save calculation time, the size of the model is 5 m × 5 m × 2 m. The diameter of the blast hole is 42 mm, the depth of the blast hole is 1.4 m, and the diameter of the cartridge is 32 mm. The first joint is 1 m away from the center of the blast hole, and the two joints are separated by 1 m. The keyword *DEFINE is used to define the load curve CURVE, 0–1.5 MPa is loaded, the keyword *INTERFACE is used to output the DYINA file with in situ stress, the original k file is replaced, and 1.5 MPa of stress is applied on the top and side surfaces of the model. The rock is defined as a solid and the Holmquist–Johnson–Cook (HJC) constitutive model is adopted, with the parameters shown in Table 3. The joint material parameters are shown in Table 4. Explosives and air are defined as fluids, and the parameters are shown in Tables 5 and 6. The fluid is divided into meshes using common nodes, and the solid and the fluid are connected using fluid–solid coupling. Except for the free surface, all other surfaces are set to no reflection boundary conditions. Four monitoring points (*A*, *B*, *C*, and *D*) were arranged at 0.6 m, 1.2 m, 1.8 m, and 2.4 m from the center of the blast hole to record the stress attenuation pattern during the blasting. The numerical simulation results and the calculation results of Eq. (15) are compared and analyzed, as shown in Fig. 9d –f.

**Table 3:** Parameters of 60° slate HJC constitutive model.

Basic mechanical parameters		Strength parameters		Damage parameters		Pressure parameters	
*ρ*	2752 kg/m^3^	*A*	0.33	*D*_*1*_	0.026	*P*_*c*_	18.47 MPa
*f*_*c*_	55.4 MPa	*B*	0.5765	*D*_*2*_	1.0	*μ*_*c*_	7.3 × 10^–4^
*G*	15.212 GPa	*C*	0.000624	*EF*_*MIN*_	0.01	*P*_*l*_	2.0 GPa
*T*	12.03 MPa	*N*	0.413	*F*_*S*_	0.004	*μ*_*l*_	0.054
		*SFMAX*	20.0			*K*_*1*_	39 GPa
		*EPSO*	1.0			*K*_*2*_	-223 GPa
						*K*_*3*_	550 GPa

**Table 4:** Basic parameters of joint materials^41^.

Density/kg m^−3^	Elastic modulus/GPa	Poisson's ratio	Yield stress/MPa	Shear modulus/GPa
2500	30	0.3	4	11.5

**Table 5:** Basic parameters of No. 2 rock emulsion explosive^42^.

Density/kg m^−3^	Detonation velocity/m s^−1^	*A*/GPa	*B*/GPa	*R*_*1*_	*R*_*2*_	$\omega$	*E*_*0*_/GPa	*V*
1240	4200	214.4	0.182	4.2	0.9	0.15	4.192	1

**Table 6:** Basic parameters of air materials^43^.

Density/kg m^−3^	*C*_*0*_	*C*_*1*_	*C*_*2*_	*C*_*3*_	*C*_*4*_	*C*_*5*_	*C*_*6*_	*E*	*V*_*0*_
1290	0	0	0	0	0.4	0.4	0	2.5e−6	1

Under the three joint inclination angles, the theoretical calculation results of the stress wave attenuation are consistent with the numerical simulation results, which verifies the correctness of the explosion stress wave attenuation formula proposed in this paper. With the increase in blast center distance, the radial stress wave peak value gradually decreases. During the propagation from point *A* to point *D*, the attenuation amplitude of the peak value of the radial stress wave is 60%. The attenuation amplitude of the radial stress wave passing through the first joint is 30%, and the attenuation amplitude when passing through the second joint is 18%. The explosion stress wave occurs with multiple reflections at the joint, resulting in the attenuation of the peak value of the radial stress wave. The first joint is close to the explosion source, and the attenuation amplitude is large. In the calculation results of Eq. (15), the peak values of radial stress waves at point *A* under the three joint inclination angles are different, because all the Eq. (15) calculation results have considered the influence of joints.

## Numerical simulation

According to the derivation of the peripheral hole spacing calculation formula under the action of in situ stress and joints in section "Determination of the peripheral hole spacing", combined with the blasting parameters of the Bayueshan Tunnel, the values of the peripheral hole spacing *D*_*P*_ and the minimum resistance line *W* are determined. The average burial depth of the Bayueshan Tunnel study section is 56 m, the slate density is 2752 kg/m^3^, and *σ*_*V*_ = *σ*_*H*_ = *ρgh* = 1.5 MPa. The physical attenuation coefficient $\alpha = 0.56224$ is calculated by Eq. (10). The No. 2 rock emulsion explosive is used for the on-site blasting, the density of the explosive is 1.24 g/cm^3^, the detonation speed is *D* = 4200 m/s, the diameter of the blast hole is 42 mm, and the diameter of the cartridge is 32 mm. Peripheral holes are equipped with 1.5 cartridges, the charge length *l*_*c*_ is 0.45 m, and the blast hole length *l*_*b*_ is 1.4 m. Calculated using Eq. (14), the impact stress of the blast hole wall after the explosion is *P*_*0*_ = 177.6 MPa. It can be seen from Table 2 that the joint correction coefficient *K* of the double-joint model is 0.711 when the confining pressure is 1.5 MPa and the joint inclination angle is 60°. In engineering blasting, it can be considered that the relationship between the dynamic Poisson's ratio *μ*_*d*_ and the static Poisson's ratio *μ* is *μ*_*d*_ = 0.8*μ*^38^. The calculated dynamic Poisson's ratio *μ*_*d*_ is 0.22, and the lateral pressure coefficient of the rock mass is $b = \mu_{d} /\left( {1 - \mu_{d} } \right) = 0.28$. It can be seen from Table 2 that the dynamic tensile strength of 60° slate is 12.03 MPa. Calculated using Eq. (17), the radius of the crack ring is *R* = 18.1 cm. Using Eq. (19), *P*_*a*_ is calculated as 3.7774 GPa, and *P*_*b*_ = 24.6 MPa. Equation (20) calculates that the peripheral hole distance *D*_*P*_ = 45 cm under the action of in situ stress and joints.

To verify that 45 cm is the optimal value of the peripheral hole spacing around the research section of the Bayueshan Tunnel, the peripheral hole distances *D*_*P*_ are set as 40 cm, 45 cm, 50 cm, and 55 cm, respectively, and the corresponding minimum resistance lines *W* are 50 cm, 56 cm, 62 cm, and 68 cm, respectively. By selecting the material constitutive model and determining the model parameters, the numerical analysis model of Bayueshan Tunnel blasting is established.

### Constitutive model selection and parameter determination

1. 60° slate constitutive model and parameter determination.

The HJC (Holmquist–Johnson–Cook) constitutive model is a rate-dependent constitutive model proposed by Holmquist, Johnson, and Cook to solve the problem of the large deformation of concrete under a high strain rate and a high pressure load^39^, and is widely used in the dynamic analysis of rock materials. The HJC constitutive model totally includes 21 parameters: basic mechanical parameters—*R*_*0*_, *f*_*c*_, *G*, and *T*; strength parameters—*A*, *B*, *C*, *N*, *SFMAX*, and *EPSO*; damage parameters—*D*_*1*_*, D*_*2*_, *EF*_*MIN*_, and *F*_*S*_; and pressure parameters—*P*_*c*_, $\mu_{c}$, *P*_*l*_, $\mu_{l}$, *K*_*1*_*, K*_*2*_*,* and *K*_*3*_.

The basic mechanical parameter *R*_*0*_ is taken as 2752 kg/m^3^, *f*_*c*_ is taken as the bidirectional compressive strength of 55.4 MPa, $G = E/2\left( {1 + \mu } \right) = 15.212\;\;{\text{GPa}}$, $K = E/3(1 - 2\mu ) = 25.35\;\;{\text{GPa}}$, and *T* takes 12.03 MPa.

**Figure 10:**
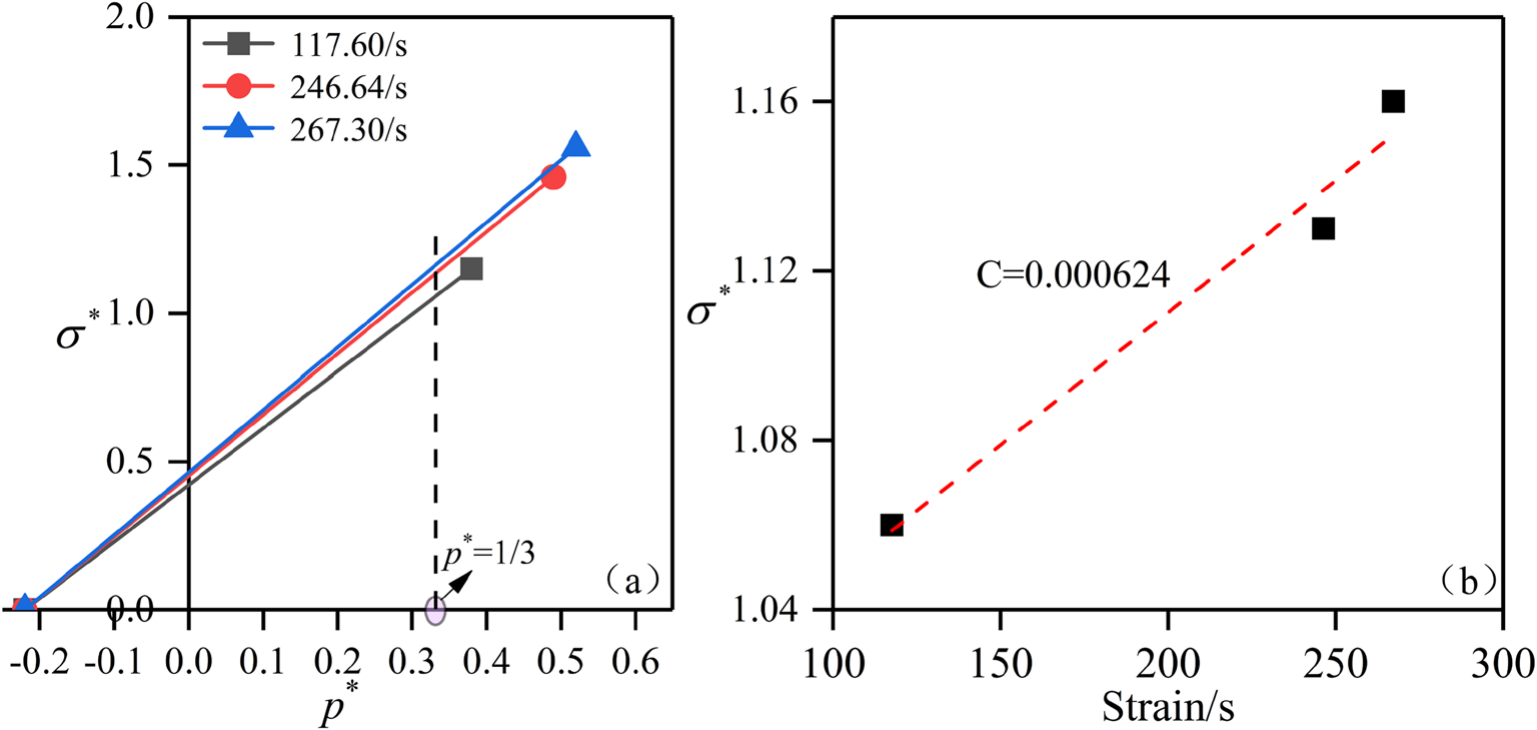
Determination of the strain rate influence coefficient *C*. Relationships between: (**a**) characteristic equivalent stress and characteristic hydrostatic pressure under different strain rates; and (**b**) characteristic equivalent stress and strain rate.

Take the 0.15 MPa impact air pressure test result in Fig. 5a as an example. To eliminate the influence of hydrostatic pressure on the dynamic strength of limestone, starting from the characteristic tensile strength $T^{*} = T/f_{c}$, straight lines are drawn through the data points of equivalent strength under different strain rates, and a straight line is drawn perpendicular to the horizontal axis at the constant characteristic hydrostatic pressure P* = 1/3. The intersection point of the straight lines at different slopes represents the characteristic equivalent stress of 60° jointed slate under different strain rates, as shown in Fig. 10a. By fitting the data points of the characteristic equivalent stress under different strain rates using the straight-line equation, the strain rate influence coefficient *C* = 0.000624 was obtained, as shown in Fig. 10b.

According to the triaxial compression test results of 60° slate, the cohesion *c* = 18.4 MPa was calculated. The characterized cohesion strength is *A* = *c*/(1 + *C*ln10^−4^) *f*_*c*_, and by substituting the value of the strain rate influence coefficient *C*, the characteristic cohesion strength *A* = 0.33 can be obtained. According to $\sigma^{*} = \left( {\sigma_{1} - \sigma_{3} } \right)/f_{c}$ and $p^{*} = \left( {2\sigma_{1} + \sigma_{3} } \right)/3f_{c}$, $\sigma^{*} - P^{*}$ curves are drawn, and the fitting can be obtained such that the values of parameters *B* and *N* are 0.5765 and 0.413, respectively. *SFMAX* and *EPSO* are, respectively, 20.0 and 1.0 according to the literature^40^.

For the damage parameters, $D_{1} = 0.01/(1/6 + T^{*} ) = 0.026$ and *D*_*2*_ takes a constant value of 1.0. According to the literature^40^, *EF*_*MIN*_ and *F*_*S*_ are taken as 0.01 and 0.004, respectively.

The pressure parameter is *P*_*c*_ = *f*_*c*_/3 = 55.4/3 = 18.47 MPa. *μ*_*c*_ = *P*_*c*_/*K* = 7.3 × 10^–4^, $\mu_{l}$ is obtained from $\mu_{l} = \rho_{g} /\rho_{0} - 1 = 0.054$, where $\rho_{g}$ is the compacted density, which is 2900 kg/m^3^. The parameters *P*_*l*_, *K*_*1*_, *K*_*2*_, and *K*_*3*_ are non-sensitive parameters, and the research results of this paper refer to the literature^40^ for their values. The parameters of the 60°slate constitutive model are shown in Table 3.

2. Joint constitutive model and parameter determination.

The No. 003 material model *MAT_PLASTIC_KINEMATI is selected as the material constitutive model of the joint, and its basic parameters are shown in Table 4.

3. Explosive constitutive model and parameter determination.

The material constitutive model of the explosive material selects the No. 008 material model *MAT_HIGH_EXPLOSIVE_BURN, and the pressure, volume, and energy characteristics of the explosive during the explosion process are simulated by defining the explosive detonation parameters in the explosive state equation *EOS_JWL. The JWL state equation is:

21 $$ P = A\left( {1 - \frac{\omega }{{R_{1} V}}} \right)e^{{ - R_{1} V}} + B\left( {1 - \frac{\omega }{{R_{2} V}}} \right)e^{{ - R_{2} V}} + \frac{{\omega E_{0} }}{V} $$

where *P* is the unit detonation pressure; *V* represents the initial relative volume; *E*_*0*_ stands for the initial specific internal energy; and *A*, *B*, *R*_*1*_, *R*_*2*_, and $\omega$ are the basic parameters of the material.

The No. 2 rock emulsion explosive is used for blasting the Bayueshan Tunnel, and its basic parameters are shown in Table 5.

4. Air constitutive model and parameter determination.

The air material constitutive model selects the No. 009 material model *MAT_NULL, and the EOS_LINEAR_POLYNOMIAL state equation is:

22 $$ P = C_{0} + C_{1} \mu + C_{2} \mu^{2} + C_{3} \mu^{3} + (C_{4} + C_{5} \mu + C_{6} \mu^{2} )E $$

where *E* is the internal energy of the material; and *C*_*0*_, *C*_*1*_, *C*_*2*_, *C*_*3*_, *C*_*4*_, *C*_*5*_, and *C*_*6*_ represent the coefficients of the state equation. The basic parameters are shown in Table 6.

5. Determination of the constitutive model and parameters of the clay.

The No. 005 material model *MAT_SOIL_AND_FOAM is selected as the material constitutive model of the clay material, and its basic parameters are shown in Table 7.

**Table 7:** Basic parameters of the clay material^44^.

Density/kg m^−3^	Elastic modulus/GPa	*μ*
1850	1.6e–4	0.3

### Model establishment

**Figure 11:**
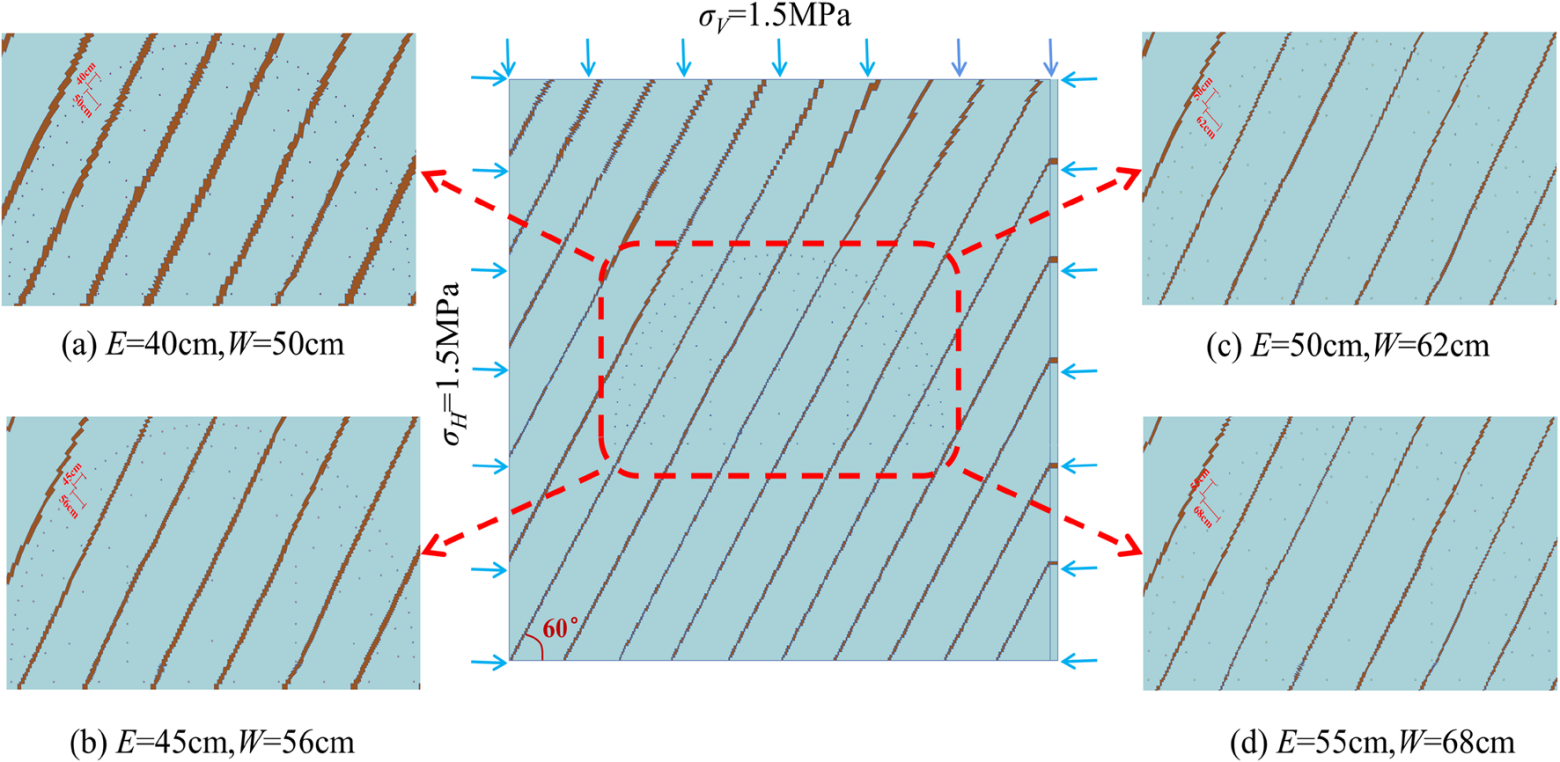
Three-dimensional numerical analysis model. (**a**) *E = *40 cm; (**b**) *E = *45 cm; (**c**) *E = *50 cm; (**d**) *E = *55 cm.

The research section of the Bayueshan Tunnel adopts the upper and lower bench blasting construction method. In this paper, numerical analysis models of upper bench blasting are established to discuss the influence of peripheral hole spacing on the damage depth and peak vibration velocity of the retained rock mass. LS-PREPOST software is used to establish a three-dimensional numerical multi-joint model, when the in situ stress is 1.5 MPa, the joint angle is 60°, and the physical attenuation coefficient *α* and the joint correction coefficient *K* are 0.56224 and 0.711, respectively. The model size is 20 m × 20 m × 2 m, and the mesh is divided by mapping to achieve common nodes between fluids and between solids. The rock mass, joints, explosives, air, and clay use the material models and parameters in Tables 3, 4, 5, 6 and 7. Stress of 1.5 MPa is applied to the top and side surfaces of the model. To avoid the influence of reflected waves caused by artificial boundaries on the calculation results, the tunnel surface is set as free boundary conditions, and the other planes are set as non-reflection boundary conditions. The three-dimensional numerical analysis model is shown in Fig. 11, and the parameters of each blast hole in the model are shown in Table 8.

**Table 8:** Blast hole parameters.

Blasthole type	Number of holes	Hole spacing/cm	Hole depth/m	Charge/kg	Detonation sequence
Cut hole	12	0.6	2.0	1.2	MS1
Bomb hole	2	1.5	1.4	0.9	MS3
The first row of auxiliary holes	10	0.95	1.4	0.9	MS3
The second row of auxiliary holes	16	0.9	1.4	0.9	MS5
The third row of auxiliary holes	25	0.8	1.4	0.9	MS7
Peripheral hole	55	40	1.4	0.45	MS9
	50	45			
	45	50			
	40	55			
Bottom hole	10	1.2	1.4	1.2	MS9

### Analysis of results

#### Retained rock mass damage depth

**Figure 12:**
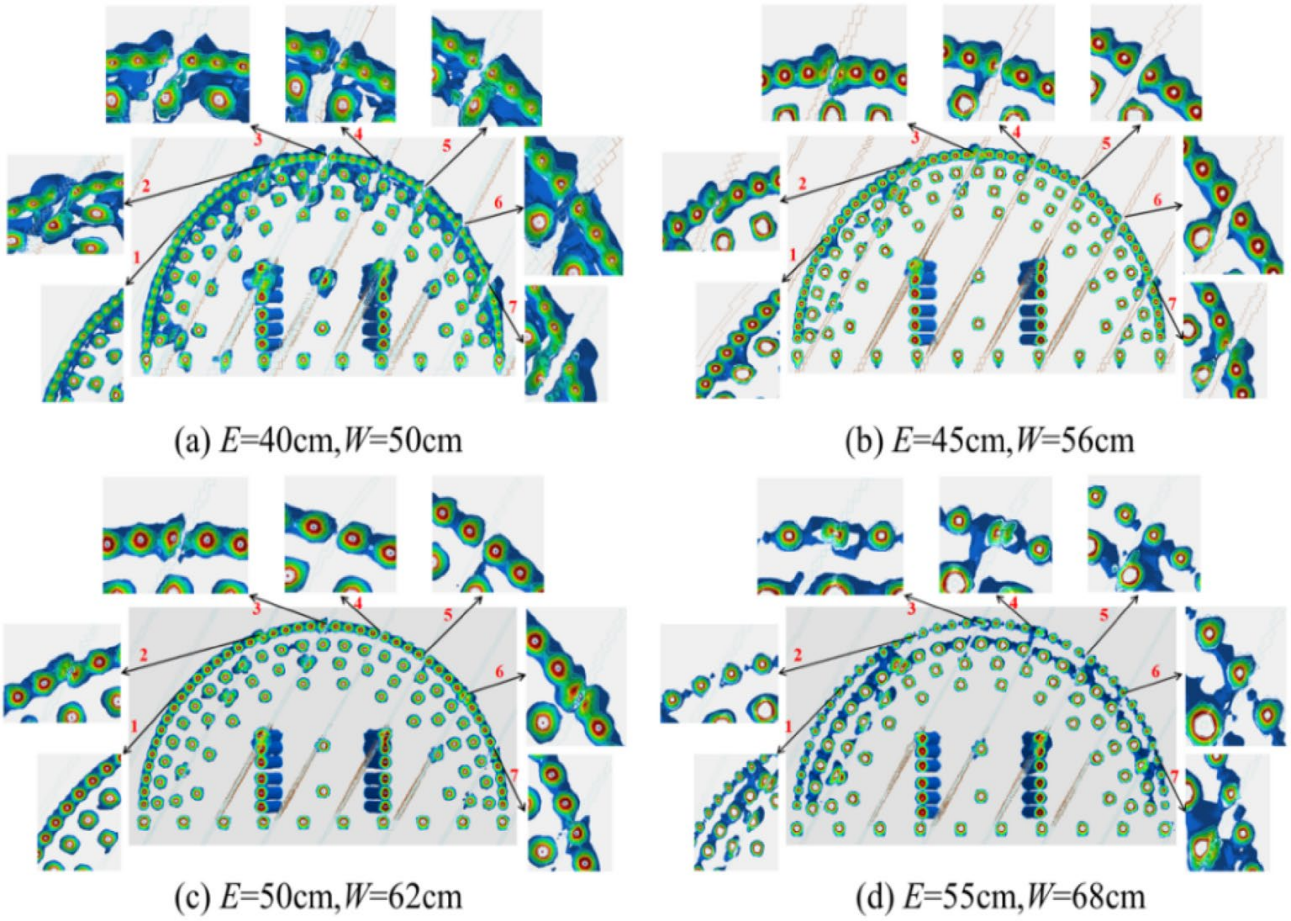
Cloud map of accumulated damage of retained rock mass. (**a**) *E* = 40 cm; (**b**) *E* = 45 cm; (**c**) *E* = 50 cm; (**d**) *E* = 55 cm.

**Figure 13:**
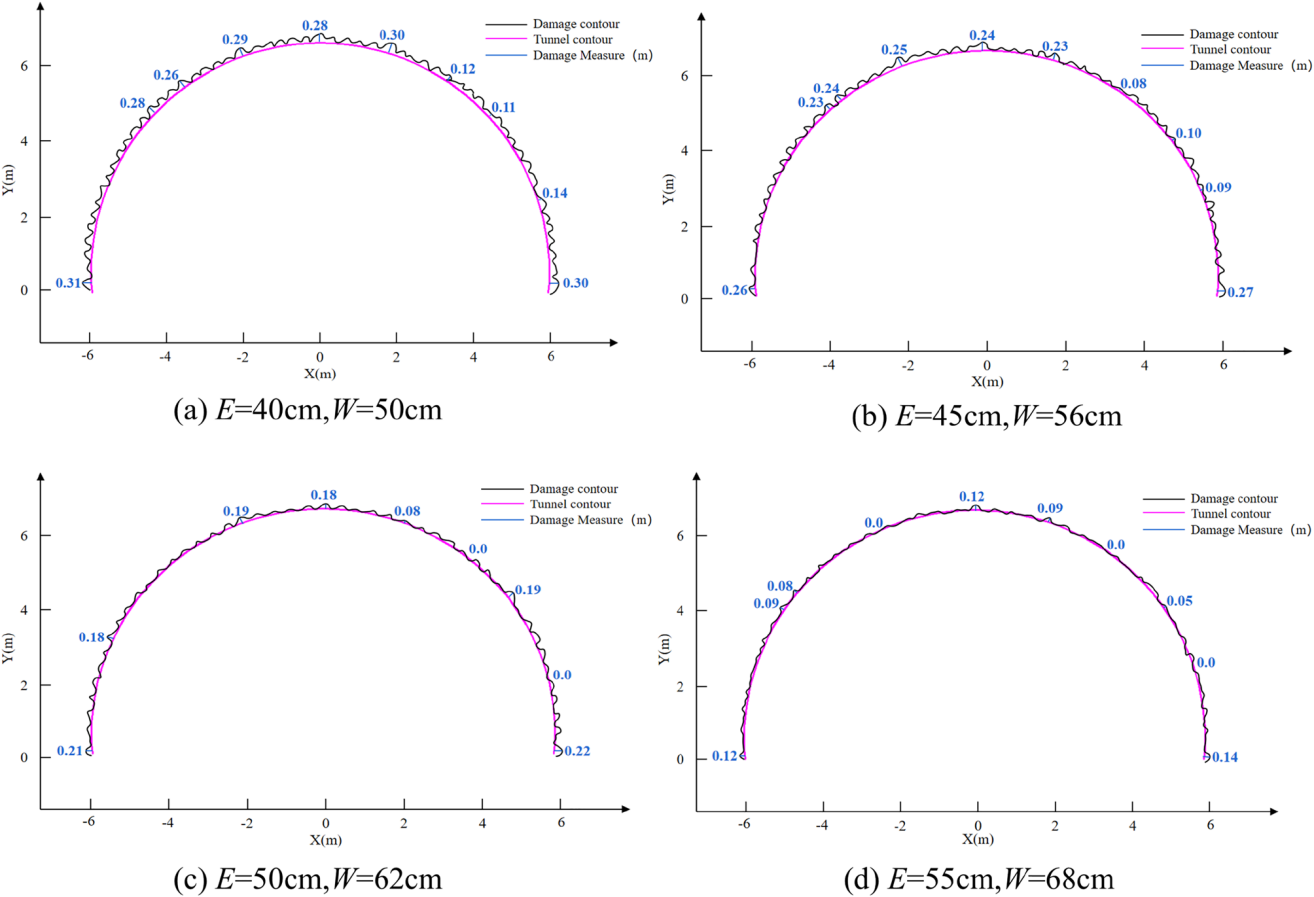
Damage depth contour. (**a**) *E* = 40 cm; (**b**) *E* = 45 cm; (**c**) *E* = 50 cm; (**d**) *E* = 55 cm.

Figure 12 shows the cumulative damage cloud map of the remaining rock mass after all blasts are completed, with the damage levels decreasing from red to green to blue. The cumulative damage cloud map shows that the damage depth gradually degenerates from the explosion center to the outer free surface. Wang et al.^45^ showed that the red damage depth line can be used to estimate the over-excavation and under-excavation values after tunnel blasting. Therefore, the red damage depth contour lines under blasting with different peripheral hole distances are drawn, as shown in Fig. 13. It can be seen from Fig. 13 that the remaining rock mass after blasting with different peripheral hole spacings is damaged to different degrees, resulting in different levels of over-excavation and under-excavation. When the peripheral hole spacing is 55 cm and 50 cm, although the damage depth of the remaining rock mass in each part is small, there are different degrees of under-excavation. Under-excavation will affect the steel arch erection process, and in severe cases, additional explosives are required, increasing the operating cost. The main reason for this phenomenon is that the distance between the peripheral holes is too far, the explosion stress wave cannot produce the superposition enhancement effect, and no through cracks are formed between the blast holes. When the peripheral hole spacing is 45 cm, the damage depth of the remaining rock mass at each part is small, and there is no under-excavation. When the peripheral hole spacing is 40 cm, the damage depth of the remaining rock mass in each part is slightly greater than 45 cm, and there is also no under-excavation. This is because the explosion stress wave produces a large superposition enhancement effect between the blast holes, and more residual energy causes more damage to the retained rock mass. When the peripheral hole distance is 40 cm, the drilling workload increases and the operating cost increases. Therefore, from the perspective of retaining the damage depth of the rock mass, the optimal value of peripheral hole spacing is 45 cm, which is consistent with the calculation result of Eq. (20).

#### Peak vibration velocity (PPV)

**Figure 14:**
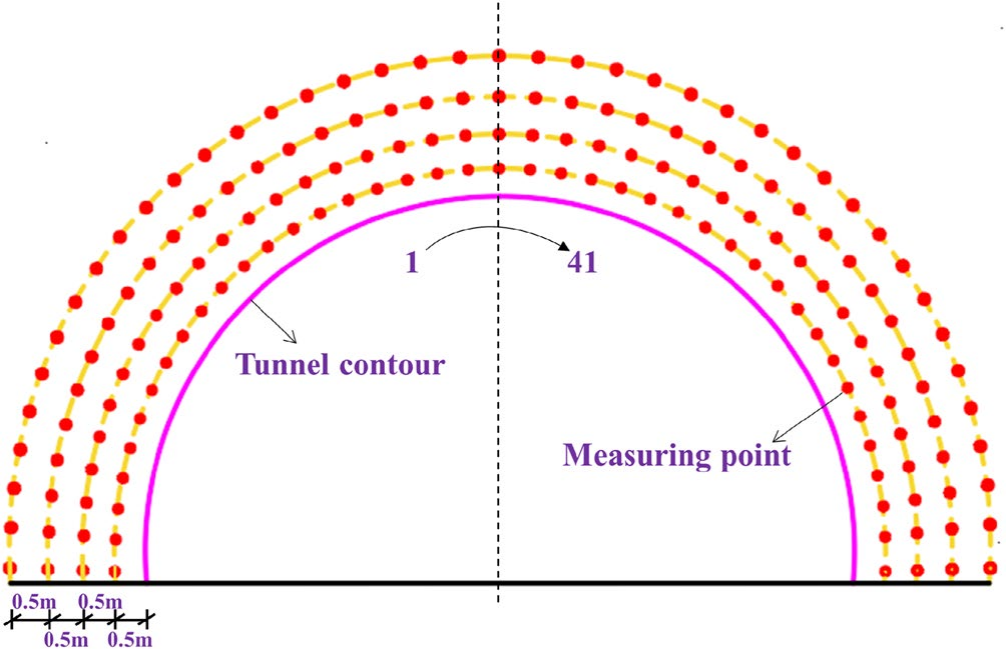
Layout of measuring points.

**Figure 15:**
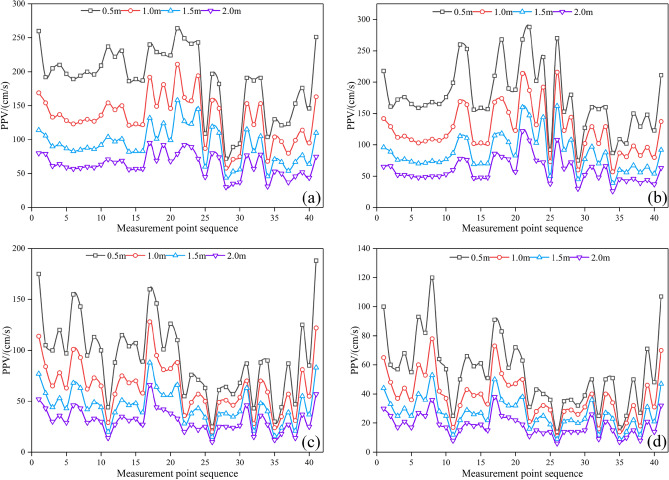
PPV curve. (**a**) *E* = 40 cm; (**b**) *E* = 45 cm; (**c**) *E* = 50 cm; (**d**) *E* = 55 cm.

The *PPV* method is the most commonly used method for the actual measurement of surrounding rock damage in the field^46–48^. Four groups of *PPV* measuring points were arranged at 0.5 m, 1.0 m, 1.5 m, and 2.0 m away from the tunnel outline, with 41 measuring points in each group evenly distributed around the tunnel outline, as shown in Fig. 14. The *PPV* curves of different distances are shown in Fig. 15. The average *PPV* values of each group when the peripheral hole distances are 40 cm, 45 cm, 50 cm, and 55 cm are shown in Table 9. As the distance from the tunnel contour increases, the average *PPV* value gradually decreases, and the attenuation amplitude is related to the distribution of joints. With the increase in the peripheral hole spacing, the average *PPV* value of each group gradually decreases. Compared with the average *PPV* value when the peripheral hole spacing is 40 cm, the average *PPV* value attenuation amplitude is smaller when the peripheral hole spacing is 45 cm, and the average *PPV* value attenuation amplitude is larger when the peripheral hole spacing is 50 cm and 55 cm. According to the *PPV* damage criterion of jointed rock mass^49^, for hard rock, the threshold is 70 cm/s. When the peripheral hole spacing is 40 cm, 45 cm, 50 cm, and 55 cm, the damage range is 183 cm, 167 cm, 115 cm, and 62 cm, respectively. The greater the peak vibration velocity *PPV*, the greater the damage depth. Combined with the analysis results in section "Retained rock mass damage depth", it can be seen that when the peripheral hole spacing is 40 cm, the average *PPV* value is larger, the damage depth is larger, and it is easy to cause greater over-excavation. When the peripheral hole spacing is 50 cm or 55 cm, the average PPV value is small, the damage depth is shallow, and the phenomenon of under-excavation occurs easily. Therefore, considering the peak vibration speed, the optimal value for the peripheral hole distance is 45 cm.

**Table 9:** Average *PPV* value.

Peripheral hole spacing/cm	Average *PPV* value/cm·s^−1^			
	Group 1	Group 2	Group 3	Group 4
40	189	132	92	63
45	175	123	87	60
50	104	75	56	41
55	75	58	42	28

## Discussion

### Field blasting test

**Figure 16:**
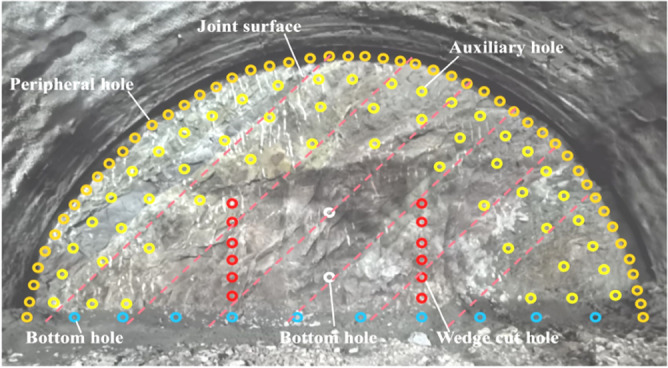
Layout of hole mesh for field blasting test.

Based on the theoretical derivation results in section "Determination of the peripheral hole spacing" and the numerical analysis results in section "Numerical simulation", the peripheral hole spacing *D*_*P*_ around the study section of the Bayueshan Tunnel is 45 cm, the resistance line *W* is 56 cm, the hole network layout is shown in Fig. 16, and the hole network layout parameters are shown in Table 7 (the peripheral hole distance is 45 cm).

**Figure 17:**
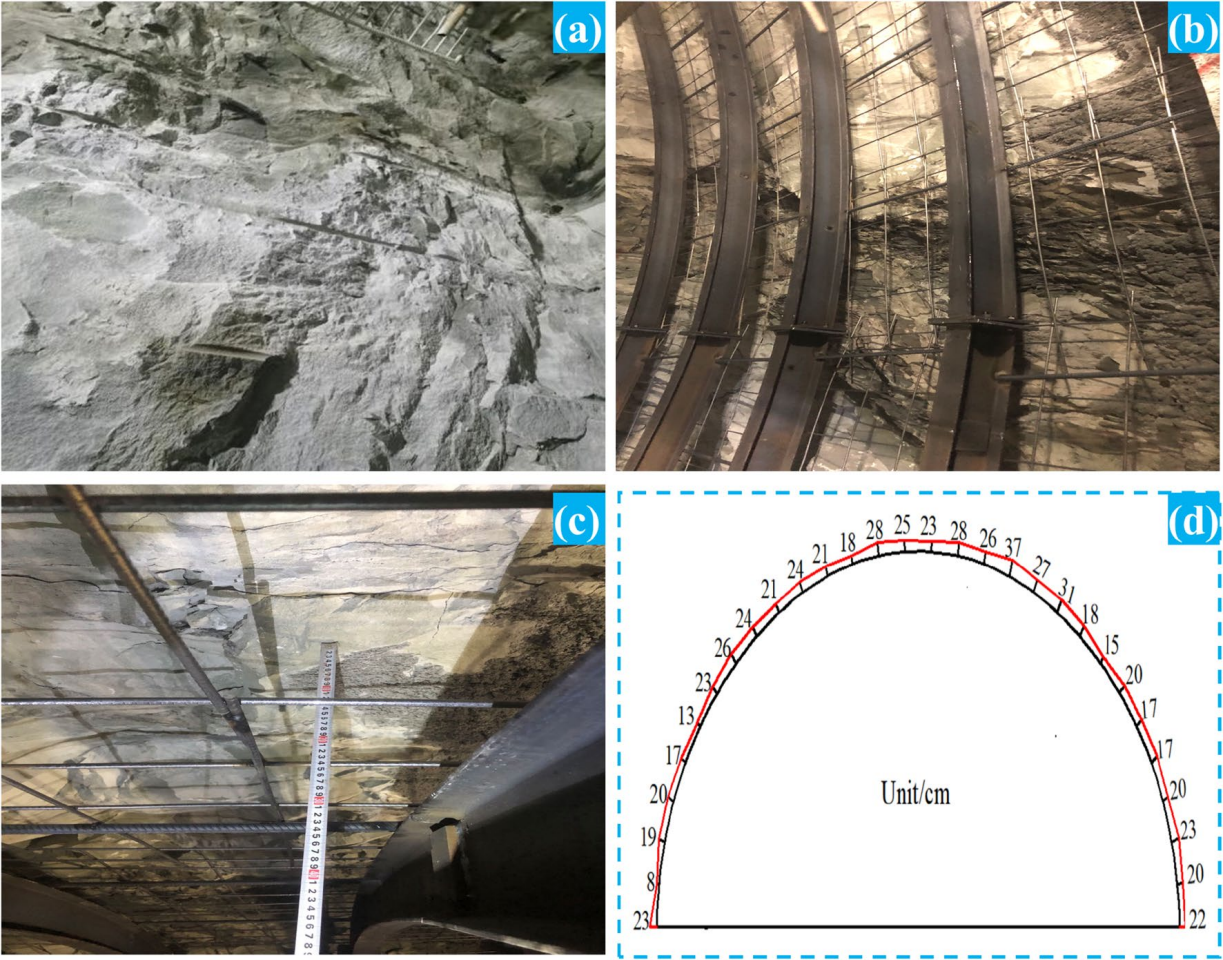
Blasting effect. (**a**) Peripheral hole residual hole; (**b**) steel arch erection effect; (**c**) and (**d**) measurement of over-excavation and under-excavation.

Three field blasting tests were carried out using the above methods and parameters. After the completion of the first blasting, the residual holes in the peripheral holes of each part are clear, as shown in Fig. 17a. The effect of over-excavation and under-excavation is shown in Fig. 17b. Measurements of the over-excavation and under-excavation for each part of the upper step are shown in Fig. 17c, d. It can be seen from the measurement results that, after the blasting of the upper steps is completed, all parts are over-excavated and there is no under-excavation. The average over-excavation is 21.8 cm, the over-excavation per linear meter is 5.82 m^3^, the design concrete dosage for the upper steps is 6.41 m^3^ per linear meter, and the concrete over-consumption per linear meter is 90.8%. After the completion of the second blasting, the average over-excavation is 22.1 cm, the over-excavation per linear meter is 5.78 m^3^, and the concrete over-consumption per linear meter is 90.2%. After the third blasting, the average over-excavation is 21.4 cm, the over-excavation per linear meter is 5.9 m^3^, and the concrete over-consumption per linear meter is 92%. Under the combined action of in situ stress and joints, the blasting construction is carried out using the peripheral hole layout method and hole network layout parameters proposed in this paper, the concrete excess consumption per linear meter is controlled within 100%, and the concrete excess consumption control effect is good.

### Influence of joint location

Seven representative locations of the cumulative damage cloud map were selected for zoom-in analysis, as shown in Fig. 12. Take Fig. 12b as an example for analysis. Position 1: When the line connecting the peripheral holes is parallel to the joint surface, the damage degree of a single blast hole increases, because the explosion stress wave causes multiple reflections at the joint surface, forming a superposition of stress waves. Position 2, Position 3, and Position 4: When the peripheral holes are just located on the joint surface, the explosion stress wave first propagates along the joint surface, and further expands and penetrates based on the original joint surface, resulting in serious over-excavation. Position 5, Position 6, and Position 7: When the joint surface is located between two peripheral holes, the explosion stress wave has a large attenuation effect on the joint surface, so the over-excavation phenomenon near these positions is effectively controlled. In summary, the relative position of the joint surface and the peripheral holes will have a great impact on rock mass damage, and on over-excavation and under-excavation^50,51^, which can be overcome by increasing or decreasing the charge.

## Conclusions

1. Uniaxial compression, triaxial compression, Brazilian splitting, and dynamic shock compression tests were carried out in this paper, and the static and dynamic parameters of 60° jointed slate were obtained. The HJC constitutive model was selected as the jointed slate material model, the method for determining the parameters of the HJC constitutive model was provided, and the parameters of the HJC constitutive model were determined.
2. Considering the geometric attenuation and physical attenuation of the blast stress wave, the attenuation formula of the blast stress wave under the combined action of in situ stress and joints was deduced. Using LS-PREPOST software, a three-dimensional numerical calculation model under the combined action of in situ stress and joints was established, and the radial stress wave peaks at 0.6 m, 1.2 m, 1.8 m, and 2.4 m from the center of the blast hole were monitored. These were consistent with the predicted value of the explosion stress wave attenuation formula proposed in this paper, verifying the correctness of the attenuation formula. Based on the theory of the interaction between stress waves and explosive gas, a peripheral hole spacing calculation formula was proposed that comprehensively considers the effects of in situ stress, joints, and rock mass tensile strength.
3. Combined with the geological conditions and blasting parameters of the Bayueshan Tunnel study section, it was calculated that the optimal peripheral hole spacing around the study section was 45 cm. A three-dimensional numerical analysis model was established using LS-PREPOST software. The damage depth of the retained rock mass and the peak vibration velocity (*PPV*) were compared and analyzed when the peripheral hole spacings were 40 cm, 45 cm, 50 cm, and 55 cm, respectively. The numerical simulation results verified the correctness of the peripheral hole spacing calculation formula proposed in this paper.
4. Taking the 45 cm peripheral hole spacing and the 56 cm resistance line as the standard, a field blasting test was carried out in the research section of the Bayueshan Tunnel. Under the combined action of in situ stress and joints, blasting construction was carried out using the peripheral hole layout method and the hole mesh layout parameters proposed in this paper. The test results show that the average over-excavation value of the grade IV surrounding rock was controlled at 22 cm, the concrete over-consumption per linear meter was controlled within 100%, and the concrete over-consumption control effect was good.

## Data Availability

The datasets used during the current study available from the corresponding author on reasonable request.
